# Negative Symptoms in Early-Onset Psychosis and Their Association With Antipsychotic Treatment Failure

**DOI:** 10.1093/schbul/sbx197

**Published:** 2018-01-24

**Authors:** Johnny Downs, Harry Dean, Suzannah Lechler, Nicola Sears, Rashmi Patel, Hitesh Shetty, Matthew Hotopf, Tamsin Ford, Marinos Kyriakopoulos, Covadonga M Diaz-Caneja, Celso Arango, James H MacCabe, Richard D Hayes, Laura Pina-Camacho

**Affiliations:** 1 Department of Psychological Medicine, Institute of Psychiatry Psychology and Neuroscience, King’s College London & NIHR South London and Maudsley Biomedical Research Centre, UK; 2 South London and Maudsley NHS Foundation Trust, UK; 3 Department of Child and Adolescent Psychiatry, Institute of Psychiatry Psychology and Neuroscience, King’s College London, UK; 4 Department of Psychosis Studies, Institute of Psychiatry Psychology Neuroscience, King’s College London & NIHR South London and Maudsley Biomedical Research Centre, UK; 5 University of Exeter Medical School, UK; 6 Department of Psychiatry, Icahn School of Medicine at Mount Sinai; 7 Child and Adolescent Psychiatry Department, Hospital General Universitario Gregorio Marañón, IiSGM, School of Medicine, Universidad Complutense, CIBERSAM, Spain

**Keywords:** early-onset psychosis, first-episode psychosis, negative symptoms, antipsychotic agents, treatment resistance

## Abstract

The prevalence of negative symptoms (NS) at first episode of early-onset psychosis (EOP), and their effect on psychosis prognosis is unclear. In a sample of 638 children with EOP (aged 10–17 y, 51% male), we assessed (1) the prevalence of NS at first presentation to mental health services and (2) whether NS predicted eventual development of multiple treatment failure (MTF) prior to the age of 18 (defined by initiation of a third trial of novel antipsychotic due to prior insufficient response, intolerable adverse-effects or non-adherence). Data were extracted from the electronic health records held by child inpatient and community-based services in South London, United Kingdom. Natural Language Processing tools were used to measure the presence of Marder Factor NS and antipsychotic use. The association between presenting with ≥2 NS and the development of MTF over a 5-year period was modeled using Cox regression. Out of the 638 children, 37.5% showed ≥2 NS at first presentation, and 124 (19.3%) developed MTF prior to the age of 18. The presence of NS at first episode was significantly associated with MTF (adjusted hazard ratio 1.62, 95% CI 1.07–2.46; *P* = .02) after controlling for a number of potential confounders including psychosis diagnostic classification, positive symptoms, comorbid depression, and family history of psychosis. Other factors associated with MTF included comorbid autism spectrum disorder, older age at first presentation, Black ethnicity, and family history of psychosis. In EOP, NS at first episode are prevalent and may help identify a subset of children at higher risk of responding poorly to antipsychotics.

## Introduction

Early-onset psychosis (EOP), defined as onset before age 18 years, is a severely debilitating condition associated with long-term psycho-social impairment.^1^ As a diagnostic term, EOP covers a broad range of psychiatric illness including schizophrenia spectrum, affective and other non-affective psychotic disorders.^2^ Children with EOP often show significant levels of both positive and negative symptoms (NS) and disorganized behavior. Relative to adult-onset psychosis, children and adolescents are more likely to have a background of longer durations of untreated psychosis, poor pre-morbid adjustment, and greater number of co-existing conditions, such as neurodevelopmental and substance abuse disorders.^3,4^

Compared to work examining the pathogenesis of adult and EOP, studies which examine prognostic indicators in the years following treatment initiation are relative scarce.^1^ From the research conducted, findings suggest that both a longer duration of untreated psychosis and poorer premorbid adjustment are associated with poorer recovery in EOP. Despite previous evidence from adult-onset samples supporting the influence of NS on functional outcomes and recovery, the effect of NS on the prognosis of EOP remains relatively unexplored. NS symptoms include lack of motivation, problems with social interaction or diminished emotional range, and involve a loss or deficit in normal functioning.^5,6^ They can be enduring and inherent to the core disease process (ie, primary NS), or caused by other factors such as medication side-effects, positive symptoms, concurrent depression, or limited social stimulation (ie, secondary NS).^5,6^

At present, it is difficult to assess the prognostic implications of NS at a young person’s first presentation with psychosis.^1^ In adult-onset cases, NS are reportedly present at first-episode psychosis in about 30%–50% of patients.^7,8^ They are difficult to treat and are one of the main contributors to the functional disability observed in psychotic illness.^9–15^ In EOP cases, NS are also reportedly stable over time, but little is known about the prevalence of these symptoms at first-episode.^16^ Most studies so far have focused on early-onset schizophrenia,^17–19^ which may not generalize to the heterogeneous population of young people that first present to child and adolescent early psychosis intervention services. In addition, prior research findings have been limited by small sample sizes, convenience recruitment of more severe cases, or inclusion of those more amenable to taking part in a research study.^1,4^

The digitization of mental health records across the world, presents an alternative resource for psychosis researchers who wish to study clinical issues “in vivo.”^20^ A major strength of these data is their comprehensive inclusion of the whole population of interest, and therefore providing highly generalizable results—addressing some of the limitations related to selection bias, sample size and attrition commonly found in the cohort studies described above. At present, NS research using electronic health records (EHR) has been limited. Despite a number of robust rating scales now available to assess NS in psychosis,^21–23^ they are inconsistently applied to clinical populations treated in routine practice.^24,25^

Computational linguistics or Natural Language Processing (NLP) explores how to make computer systems understand and manipulate natural language expressed in text to perform desired tasks.^26^ Phenotype algorithms using NLP within clinical text, are an emerging method of automatically classifying patients with specific diseases, symptoms and outcomes.^27^ NLP approaches can discern the meaning or semantic content of text, and using pre-specified algorithms, encode text to provide structured output for analysis. This provides considerable advantages compared to performing key word searches in EHR, especially when accurately targeting certain clinical phenotypes.^27^ For example, NLP can discern whether a key word *emotional withdrawal* in the health record refers to a patient or family member, their current or past mental state, or is simply a negated item within clinical screening. NLP approaches can use pattern recognition via statistical or machine learning methods to identify a phenotype or exposure of interest within the EHR. Parameters around accuracy can be stipulated, allowing uncertainty on whether an event or phenotype is a true positive, which can be accounted in later analysis. Investigators have largely adopted this approach in i2b2 (Informatics for Integrating Biology and the Bedside), a US consortium, based at Harvard/MIT Health Science division and Partners HealthCare System in Boston, MA.^28^

In a large naturalistic sample of children and adolescents first presenting to services with EOP, we examined the prevalence of NS recorded in the mental health record at initial contact with psychiatric services. To address the limited structured information available on NS, we used a machine-learning NLP approach, validated in adult samples, to extract NS data within the EHR. To explore NS as potential prognostic indicator, we examined whether NS at first episode predicted antipsychotic treatment failure, using a pragmatic measure of treatment failure, as defined by initiation of a third trial of novel antipsychotic (due to prior insufficient response, intolerable adverse-effects or non-adherence), which we termed multiple treatment failure (MTF).^29^ Previous work in adult-onset samples, suggests that NS characterize psychotic disorders with non-hyperdopaminergic pathophysiology,^30,31^ which is supported by clinical evidence that NS in the first-episode are associated with poorer response to antidopaminergic effects of current antipsychotic treatment.^30,32^ Therefore, we predicted that EOP patients with NS at presentation would be more likely to experience MTF. We also expected that this association would remain after taking account of potential confounders, including type of psychotic disorder, positive symptoms, family history of psychosis, comorbid depression, and additional markers of premorbid neurodevelopmental difficulties such as co-occurring autism spectrum disorders (ASD), hyperkinetic disorder and intellectual disability.

## Methods

### Study Design and Study Sample

A complete description of the study design and sample selection is provided elsewhere.^29^ In brief, the sample consisted of a clinical cohort of all those individuals with a first episode of any psychotic disorder who were referred to child and adolescent mental health services (CAMHS)—including inpatient, outpatient, and early intervention for psychosis services—in South London and Maudsley NHS Foundation Trust (SLaM), United Kingdom, from January 1, 2008 to December 31, 2014. Over this time, SLaM delivered all aspects of inpatient and community-based child mental healthcare to approximately 250000 children residing in 4 London boroughs, and specialist provision to children resident outside the boroughs where local area services (such as inpatient facilities) were unavailable. Most children experiencing a psychotic disorder within the SLaM catchment area of South London were likely to present to SLaM services and included in this study: the private sector has very limited involvement in child mental health within the area, and children with psychosis, relative to adults, usually come to the attention of services relatively early.^33^

The data were extracted using the Clinical Record Interactive Search (CRIS) application: a de-identified record database containing the EHR of over 34400 child and adolescent cases held at the UK National Institute for Health Research (NIHR) Biomedical Research Centre (BRC) for Mental Health.^34,35^ Data from structured text fields was extracted and missing structured data was supplemented by NLP tools (Generalised Architecture for Text Engineering [GATE]^36^ and TextHunter^37^) which code “free text” from the EHR (ie, progress notes, mental state assessments, discharge summaries, outpatient correspondence). The CRIS resource was an approved as anonymized data resource for secondary analysis by Oxfordshire Research Ethics Committee C (08/H0606/71+5). This study was approved under NIHR BRC CRIS oversight committee (ref: CRIS 14–095).

### Inclusion Criteria

Inclusion criteria for participants were: (1) age 10–17 years at the time of first presentation to CAMHS (owing to ethical considerations and risk of statistical disclosure, we did not include children who were under the age of 10 y); (2) at least one “clinically relevant” psychotic disorder diagnosis, based on clinician judgment after comprehensive diagnostic interviews and identified from either clinician-recorded structured fields (ICD-10 codes F20-F29, F30-31, F32.3, F33.3, F1x.5); or any free text clinician-recorded ICD-10 diagnosis of “schizophrenia,” “schizoaffective disorder,” “bipolar disorder,” “depression with psychotic symptoms,” “brief psychotic disorder,” “delusional disorder,” “shared psychotic disorder,” “drug-induced psychosis,” and “psychosis not otherwise specified (NOS),” filtered for any clinician-recorded mention of antipsychotic treatment after the psychosis diagnosis. The earliest recorded psychosis diagnosis was coded as the first-episode diagnosis. For reporting purposes, diagnoses were grouped into schizophrenia, schizoaffective disorder, bipolar disorder, psychotic depression, drug-induced psychosis, and other psychoses (including brief psychotic disorder, delusional disorder, shared psychotic disorder, and psychoses-NOS). A hand-searched review of a random sample of 100 records revealed that this identification process had a 0.98 positive predictive value (PPV) for psychosis.

**Fig. 1.**
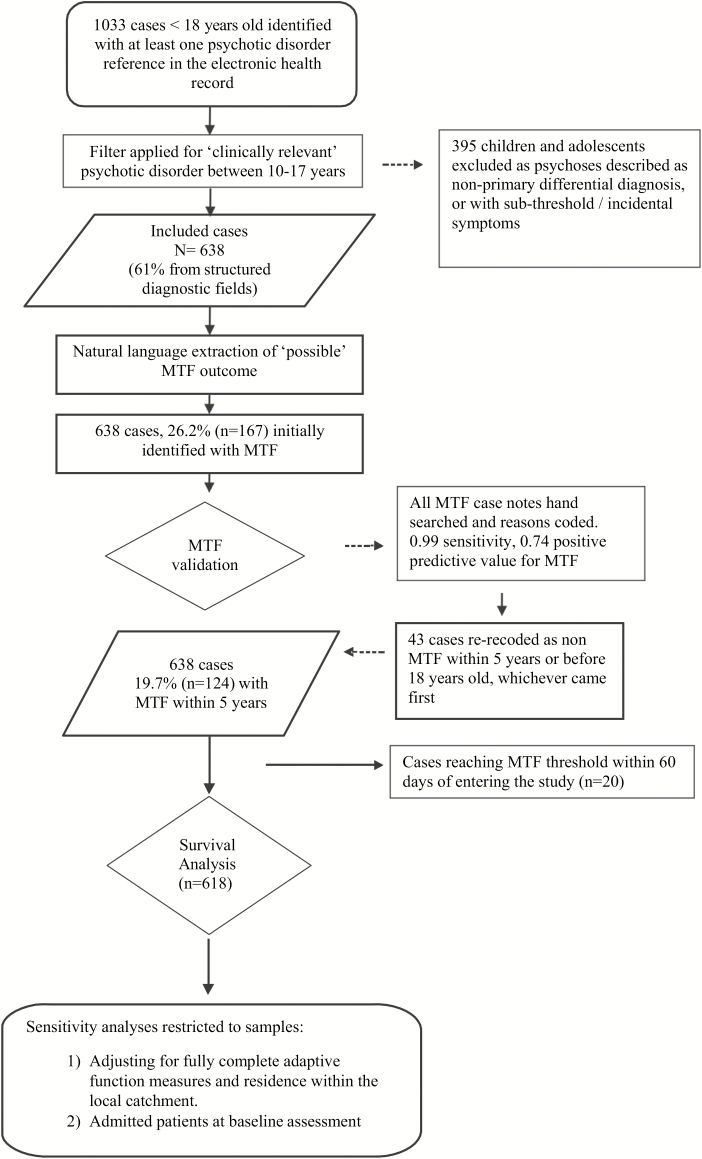
Flowchart of study selection and analysis.

Figure 1 shows the flowchart for inclusion in the study. Out of the 1033 cases initially identified with the GATE tool or through structured diagnoses, only 638 individuals met the inclusion criteria for a “clinically relevant psychotic disorder” and age 10–17 years and were therefore included, whilst 395 were excluded due to psychosis referring to non-primary/differential diagnosis or subthreshold symptoms.

### Extraction of Antipsychotic Use Data and Definition of MTF

As described elsewhere,^29^ we used a previously validated GATE application to identify regular antipsychotic prescription trials from the structured medication fields and unstructured fields in the EHR.^38,39^ Since no standard criteria for poor antipsychotic response or refractory disorder appeared suitable for EOP samples,^40,41^ a proxy was created, based on the antipsychotic effectiveness literature,^42–44^ which we termed MTF; defined as the initiation of a third trial of a novel antipsychotic due to insufficient response, intolerable adverse effects, non-adherence, or other miscellaneous reasons over a 5-year follow-up period from first presentation, or before the age of 18 years, whichever came first. Please see Downs et al^29^ for further details around the validation of the MTF outcome and reasons for discontinuation.

### Extraction of NS Data

A previously validated NLP method^8^ was used to find statements in the unstructured free-text fields of patients’ EHR which related to the presence of NS at baseline (ie, within 60 d of accepted referral). The method was based on a NLP tool called TextHunter (see Jackson et al^37^ for further details) which is a custom-built NLP software tool which interfaces with CRIS. It facilitates each of the steps involved in developing a NLP application,^27^ from identifying appropriate ontologies and supporting manual annotation, to applying and testing sophisticated text-based pattern recognition (including support vector machine learning approaches) derived from annotated training datasets.

To validate the NLP data extraction, the randomized sample of 100 cases used previously was also hand-searched for NS by a master’s level graduate in Early Intervention Psychosis studies (H.D.), blinded to MTF status. The PPV for NS subtypes ranged from 0.80 (poverty of speech) to 0.99 (mutism) and sensitivity ranged from 0.62 (poor motivation) to 0.97 (apathy). For the purposes of this study, Marder negative factor items^21,45^ from the Positive and Negative Syndrome Scale (PANSS)^23^ were used as a framework for characterizing NS (see table 1 for details). The extracted item “social isolation” was considered descriptive of both passive apathetic social withdrawal (Marder N4) and active social avoidance (Marder G16). Having mutism, poverty of speech or both items recorded on the EHR was counted as a single NS, equivalent to lack of spontaneity / flow of conversation (Marder N6). The item psychomotor retardation (equivalent to Marder G7) was dropped as an NS due to its low PPV (0.55) and sensitivity (0.65). Furthermore, the hand search of the selected 100 cases revealed that this item had a low prevalence (~5% of the sample) and always appeared acknowledged as an antipsychotic-related adverse effect (hence a secondary NS).

**Table 1.** Selection of Negative Symptoms From Electronic Health Records and Their Equivalence to the Marder Negative Factor Items Within the PANSS

Items Extracted From Electronic Health Record	PPV/Sensitivity	Marder Negative Factor Items Within the PANSS
Blunted affect	0.93/0.83	N1. Blunted affect
Emotional withdrawal	0.85/0.74	N2. Emotional withdrawal
Poor rapport	0.91/0.77	N3. Poor rapport
Social isolation	0.94/0.96	N4. Passive apathetic social withdrawal
		G16. Active social avoidance
Poverty of speech	0.80/0.73	N6. Lack of spontaneity and conversation flow
Mutism	0.99/0.94	
Psychomotor retardation (dropped^a^)	0.55/0.65	G7. Motor retardation

*Note*: PANSS, Positive and Negative Syndrome Scale; PPV, positive predictive value.

^a^Dropped from the study due to low PPV (0.55) and sensitivity (0.65) of the “free text” extraction tool, and due to its being recorded mainly as secondary negative symptom.

A composite ordinal variable, “number of NS” (range 0–5) was created by summing the total count of the extracted NS. A score of at least 2 NS was applied a priori to determine the presence or absence of NS for analysis. Individuals were categorized as having an NS or non-NS profile using the ≥2 Marder Factor NS cut-off. This was consistent with previous work that used the 2-symptom cut-off to describe deficit syndromes in schizophrenia (ie, primary, enduring NS).^8,13^

### Extraction of Other Clinical and Demographic Data

A number of demographic variables and clinical data within 60 days of study entry (ie, after accepted referral) were also extracted from the health record. Age at referral for first-episode psychosis, gender, ethnicity (according to categories defined by the UK Office for National Statistics), and index of neighborhood deprivation for the main caregiver residence were extracted.^46^ Data on positive symptom severity around first presentation were extracted using TextHunter (see Jackson et al. 2017^47^ for validation metrics) which provided the total number of items in the EHR referencing positive symptoms of psychosis including delusions, persecutory and paranoid ideation, and hallucinations. Based on the total number of items referenced, individuals were then categorized into quartiles. As an additional index of severity we coded inpatient status and the Children’s Global Assessment Scores (CGAS),^48^ respectively at study entry.^29^ Data on ICD-10 comorbid neuropsychiatric disorders which can be subsumed under the DSM-5 category of ASD (F84.0, F84.1, F84.5, F84.9), hyperkinetic disorder (F90.0, F90.1, F90.2, F90.8, F90.9), major depressive disorder (F32-33), and intellectual disability (F70-79), were also extracted from free text and structured fields as previously described.^29^ TextHunter also retrieved positive mentions of substance misuse around first presentation, with validation metrics (PPV) for the following Cannabis (0.70), Cocaine or crack (0.78), Amphetamine (0.76), and 3,4- Methylenedioxymethamphetamine (MDMA, 0.88); a binary “any use” variable was created for each substance type. Using the GATE tool, we also built a rules-based NLP application which coded absence/presence of a 1^st^ degree relative with psychosis (defined as any of the study inclusion terms for psychosis but affecting parents or full siblings). Validation of this NLP approach was conducted against clinician review (JD & LP) of all patient notes from 96 randomly selected EOP cases (PPV 0.91, recall 0.73).

### Statistical Analyses

All analyses were conducted using STATA (Version 13). The prevalence of individuals meeting ≥2 threshold NS, and the total number of NS items was calculated. Logistic regression was used to examine the demographic and baseline clinical association with ≥2 NS profiles.

To examine the prospective association between baseline demographic, clinical exposures and MTF outcome, we excluded children who had MTF within the 60-day baseline period (*n* = 20). Kaplan–Meier curves were used to illustrate survival over time (probability of non-development of MTF), comparing those who were and were not presenting with ≥2 NS at baseline. After checking proportional hazards assumptions, we used a Cox regression to model the association between this baseline NS profile and MTF over a 5-year follow-up period from first presentation, or before the age of 18 years, whichever came first. The first model examined the crude effect of NS alone on MTF. Subsequent models were constructed adding potential socio-demographic, and clinical confounders. As sampling bias towards more severe cases could affect the external validity of the findings, sensitivity analyses were conducted to (1) adjust the aforementioned models by adaptive function (CGAS) measures at first presentation and local catchment area residence status; (2) restrict to patients who were inpatients at baseline assessment.

## Results

### Demographic and Clinical Characteristics of the Sample

Demographic and clinical characteristics of the 638 patients included (124 [19.3%] of whom developed MTF over time) and of the NS subgroup are presented in table 2.

**Table 2.** Comparison Between Young People With Early-Onset Psychosis at First Presentation With and Without ≥ 2 NS Documented

Sample Characteristics	Non-NS Group (*n* = 399)	NS Group (*n* = 239)	OR; *P-value*
MTF status, *N* (%)	59 (14.8)	65 (27.2)	2.15 (1.45–3.20)***
Gender, female, *N* (%)	192 (48.1)	117 (48.9)	1.03 (0.75–1.42)
Age at referral (mean, SD)	15.4 (1.9)	15.9 (1.9)	1.17 (1.06–1.28)***
Age of reaching MTF (mean, SD)	16.5 (1.3)	16.0 (0.19)	0.79 (0.61–1.04)
Duration of follow-up (d), mean (SD)	721.4 (529.9)	590.5 (458.0)	0.995 (0.991–0.998)**
Ethnicity, *N* (%)			
White	204 (51.1)	93 (38.9)	Reference
Black	113 (28.3)	96 (40.2)	1.86 (1.29–2.67)
Asian	18 (4.5)	21 (8.8)	2.56 (1.30–5.03)
Mixed	47(11.8)	27(11.3)	1.26 (0.74–2.15)
Not Stated	17 (4.3)	2 (0.8)	0.25 (0.06–1.14)
Neighborhood characteristics, *N* (%)^a^			
1^st^ (least deprived)	104 (27.1)	61 (25.9)	Reference
2^nd^	90 (23.4)	62 (26.4)	1.17 (0.75–1.42)
3^rd^	94 (24.5)	57 (24.3)	1.03 (0.66–1.63)
4^th^ (most deprived)	96 (25.0)	55 (23.4)	0.98 (0.62–1.54)
First ICD-10 psychosis diagnosis, *N* (%)			
Other psychoses^b^	63 (15.8)	43 (17.9)	Reference
Bipolar disorder / F30, F31	31 (7.8)	11 (4.7)	0.57 (0.24–1.15)
Drug-induced psychosis / F1x.x5	29 (7.3)	10 (4.2)	0.51 (0.22–1.14)
Schizophrenia / F20	222 (55.6)	143 (59.8)	0.94 (0.61–1.46)
Schizoaffective / F25	11 (2.8)	6 (2.5)	0.80 (0.27–2.32)
Psychotic depression / F32.3, F33.3	43 (10.8)	26 (10.9)	0.89 (0.47–1.65)
Comorbid neuropsychiatric disorders, *N* (%)			
Autism spectrum disorder	75 (18.8)	39 (16.3)	0.84 (0.55–1.29)
Hyperkinetic disorder	33 (8.3)	7 (2.9)	0.33 (0.15–0.77)**
Intellectual disability	43 (10.8)	22 (9.2)	0.84 (0.49–1.44)
Major depressive disorder	108 (27.1)	66 (27.6)	1.03 (0.72–1.48)
First degree relative with psychotic disorder	86 (21.6)	51 (21.3)	0.99 (0.67–1.46)
Illness severity/ functioning			
Admission at presentation, *N* (%)	90 (22.6)	170 (71.1)	8.5 (5.9–12.2)***
CGAS score (mean, SD)^c^	42.1 (15.3)	33.7 (15.4)	0.97 (0.95–0.98)***
Positive symptoms			
1^st^ (lowest quartile of symptom items recorded)	61 (15.3)	11 (4.6)	Reference
2^nd^	79 (19.8)	27 (11.3)	1.90 (0.87–4.21)
3^rd^	137 (34.3)	84 (35.2)	3.40 (1.69–6.83)***
4^th^ (highest quartile of symptoms items recorded)	122 (30.6)	117 (49.0)	5.31 (2.67–10.6)***
Substance misuse			
Cannabis	171 (42.9)	113 (39.5)	1.20 (0.87–1.65)
Cocaine or crack	65 (16.3)	39 (16.3)	1.02 (0.65–1.54)
Amphetamines	14 (3.5)	5 (2.1)	0.59 (0.21–1.65)
MDMA	12 (3.0)	4 (1.7)	0.55 (0.18–1.72)

*Note*: CGAS, Children’s Global Assessment Scale; MTF, multiple treatment failure; MDMA, 3,4- Methylenedioxymethamphetamine; NS, negative symptoms.

^a^Missing cases =19.

^b^Data available in a subsample of 384.

^c^Other Psychoses: an ICD-10 diagnosis of “brief psychotic disorder,” “delusional disorder,” “shared psychotic disorder,” or “psychosis not otherwise specified (NOS).”

**P < .01; ***P < .001; % Refers to percentages within columns, for whom information was available.

### NS Prevalence

Supplementary material 1 shows the prevalence of each NS and positive symptom, and summary statistics for each item using the manually-validated NLP extraction tool, in the total sample and the MTF subgroup. Of note, 52.4% of the MTF subgroup presented with ≥2 Marder Factor NS. The most prevalent NS in the MTF subgroup was emotional withdrawal (43.6%). The prevalence of ≥2 Marder Factor NS across diagnostic categories were as follows: schizophrenia- 39.2%, schizoaffective disorder- 35.3%, bipolar disorder- 26.1%, psychotic depression- 37.7%, drug-induced psychosis- 25.6%, and other psychoses- 40.6%.

### Reasons for Antipsychotic Discontinuation

Details on the antipsychotic treatment pathways for the 124 children who developed MTF are shown as supplementary material 2. Cases identified as having the same reason for antipsychotic discontinuation at first and second antipsychotic trials were grouped into 3 MTF “persistent reason” groups (persistent insufficient response, adverse events or non-adherence). A “variability in reasons” subgroup (ie, when reasons were different at each antipsychotic trial) was also created. The main patterns of discontinuation in the MTF group were the combination of insufficient response and adverse events (*n* = 32, 35.2%), and persistent adverse events (*n* = 19, 20.9%) over time. Children with NS profile showed higher rates of the “insufficient response-and-adverse effect” trajectory and lower rates of adherence-related trajectories relative to those with non-NS profile (supplementary material 2).

### Cox Regression Models

**Fig. 2.**
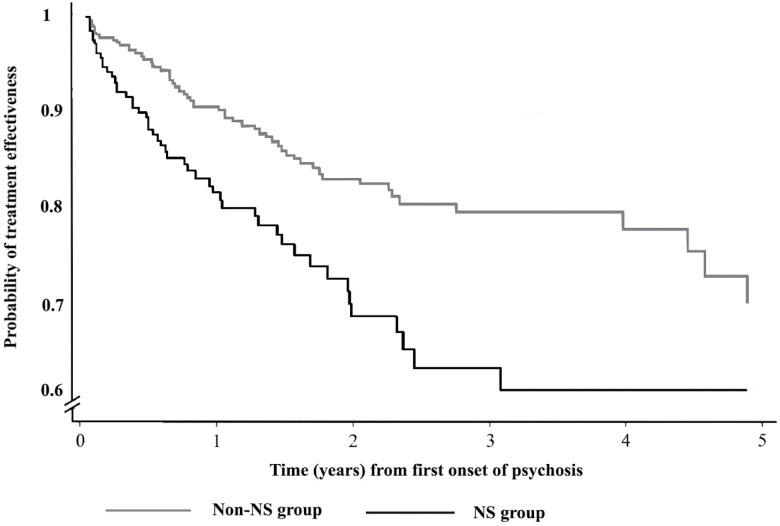
Kaplan-Meier curves displaying the survival status (probability of treatment effectiveness or non-MTF) over time of children with or without negative symptom profiles at first presentation to services.

Kaplan-Meier curves displaying the survival status (probability of treatment effectiveness or non-MTF) over time of children with or without baseline NS profiles are presented as figure 2. A log-rank test showed non-NS profile at first presentation to services displayed significantly higher survival rate (*P* < .001). Unadjusted associations using a Cox regression model between MTF outcomes and predictor variables, including NS and other co-variates, are displayed in supplementary material 3. An adjusted Cox regression model (table 3) showed that NS profile was associated with increased risk of MTF over the follow-up period (adjusted hazard ratio [aHR] 1.62, 95% CI 1.07–2.46; *P* = .02). Black ethnicity (aHR 1.78, 95% CI: 1.11–2.87; *P* = .02), older age at first presentation (aHR 1.27, 95% CI: 1.109–1.49; *P* = .002), comorbid diagnosis of ASD (aHR 1.70, 95% CI: 1.03–2.79; *P* = .04), and first degree relative with psychotic disorder (aHR 2.11, 95% CI: 1.35–3.30; *P* = .001) were also significantly associated with MTF.

**Table 3.** Cox Regression Models for the Association Between Negative Symptom Profile at First Presentation and Multiple Treatment Failure Over Time in Early-Onset Psychosis (*n* = 618)

Multiple Treatment Failure	Socio-demographic adjustment aHR (95% CI)	+ Diagnosis and Severity aHR (95% CI)	+ Substance Misuse and Family History aHR (95% CI)
≥2 baseline Marder NS	1.66 (1.12–2.42)*	1.59 (1.06–2.40)*	1.62 (1.07–2.46)*
Female gender	1.08 (0.73–1.62)	1.15 (0.76–1.74)	1.07 (0.71–1.64)
Mean age at referral (SD)	1.25 (1.09–1.45)**	1.29 (1.10–1.49)**	1.27 (1.09–1.49)**
Ethnicity, *N* (%)			
White	Reference	Reference	Reference
Black	1.95 (1.23–3.09)**	1.72 (1.08–2.76)*	1.78 (1.11–2.87)*
Asian	1.16 (0.48–2.77)	1.10 (0.46–2.67)	1.33 (0.58–3.26)
Mixed	1.51 (0.80–2.86)	1.43 (0.75–2.73)	1.63 (0.84–3.17)
Not stated	—^a^	—	—
Neighborhood characteristics, *N* (%)^a^			
1^st^ (least deprived)	Reference	Reference	Reference
2^nd^	0.60 (0.35–1.04)	0.69 (0.39–1.19)	0.66 (0.37–1.17)
3^rd^	0.55 (0.31–0.96)*	0.61 (0.35–1.08)	0.56 (0.31–0.98)
4^th^ (most deprived)	0.55 (0.31–0.97)*	0.61 (0.34–1.08)	0.62 (0.34–1.11)
First ICD-10 psychosis diagnosis, *N* (%)			
Other psychoses^b^		Reference	Reference
Bipolar disorder / F30, F31		1.57 (0.69–3.56)	1.54 (0.67–3.56)
Drug-induced psychosis / F1x.x5		0.82 (0.27–2.51)	0.92 (0.29–2.96)
Schizophrenia / F20		0.85 (0.50–1.45)	0.78 (0.45–1.32)
Schizoaffective / F25		2.42 (0.87–6.85)	2.22 (0.78–6.34)
Psychotic depression / F32.3, F33.3		1.39 (0.62–3.08)	1.15 (0.50–2.60)
Comorbid neuropsychiatric disorders, *N* (%)			
Autism spectrum disorder		1.73 (1.06–2.82)*	1.70 (1.03–2.79)*
Other neurodevelopmental disorder		0.74 (0.41–1.33)	0.68 (0.38–1.24)
Major depressive disorder		0.68 (0.39–1.15)	0.69 (0.41–1.20)
Positive symptoms			
1^st^ (lowest quartile)		Reference	Reference
2^nd^		0.89 (0.36–2.15)	0.83 (0.32–2.13)
3^rd^		1.18 (0.53–2.65)	1.09 (0.48–2.46)
4^th^ (highest quartile)		1.97 (0.92–4.21)	1.83 (0.84–3.98)
First degree relative with psychotic disorder			2.11 (1.35–3.30)**
Substance misuse			
Cannabis			1.07 (0.67–1.70)
Cocaine or crack			0.68 (0.36–1.31)
Amphetamines			1.20 (0.27–5.43)
MDMA			0.55 (0.07–4.34)

*Note*: HR, hazard ratio; MTF, multiple treatment failure; MDMA, 3,4- Methylenedioxymethamphetamine; NS, negative symptoms.

^a^Variable dropped due to 0 values in cell.

^b^See corresponding footnote table 2.

*P < .05; **P < .01.

### Sensitivity Analyses

A sensitivity analysis with adjustment for all those with complete CGAS information and residence within the local catchment area (*n* = 394), found NS profile was associated with increased risk of MTF (aHR = 1.85; 95% CI = 1.02–3.48; *P* = 03). The analyses including only those individuals who were inpatients (*n* = 260, 40.8%) at first presentation (within 60 d of accepted referral) found little change in the direction and magnitude of the association between NS and MTF (aHR = 1.63; 95% CI = 0.82–3.22; *P* = .16), although the reduced sample affected the power of the study to detect a significant association.

## Discussion

This study shows that children and adolescents with psychosis commonly present with NS, with more than one-third of the sample displaying NS at first presentation to services. Our results also show that an NS profile at first stages is a prognostic marker for antipsychotic treatment failure in children with EOP: approximately 30% of the sample with NS at baseline went on to develop MTF, representing a 2-fold increased risk from those without NS. The treatment pathway to MTF for young people with NS profiles appears to be driven by a combination of limited treatment response and emergence of intolerable adverse effects. Older age at first episode, Black ethnicity and a comorbid diagnosis of ASD are also significant predictors of MTF in our sample.

This is, to our knowledge, the largest naturalistic study of its kind to examine the prevalence of NS in EOP at first presentation to child mental health services. The study used an innovative text mining technique, adapted from an application in adult mental health records,^8^ to extract NS profiles. In our study, more than one-third of the EOP population had 2 or more NS at baseline, rates that are consistent with those reported in both child and adult-onset psychosis literature (around 30%–50%).^8,49^

This is also the first study to assess the association of NS and antipsychotic treatment failure in first-episode EOP patients. Our results, combined with findings that NS can manifest in the psychosis prodrome,^50^ suggests that NS profiles could represent a distinct phenotypic trajectory in young people with psychotic disorders. NS are possibly a marker for a distinct deviant neurodevelopmental trajectory which may be harder to treat with conventional antipsychotics and therefore result in a more impaired illness course. Although no other cohorts have been used to examine MTF as an outcome in EOP, our findings are consistent with evidence that NS are associated with poor clinical outcomes in adult and child samples, many of those using validated gold-standard instruments to measure NS (eg, the PANSS).^1,51^ Our work using text mining approaches for NS identification in large scale naturalistic samples of EOP using EHRs serves to complement the more traditional approaches using selective cohorts and intensive structured assessments, to inform prognostic indicators in clinical practice.

Several alternative psychopathological processes may be driving our findings. Higher levels of primary NS may represent a clinical phenotype for greater levels of “non-hyperdopaminergic” processes behind psychosis development and/or remission.^30,31,52^ Hence, NS may help identify a subgroup of patients with positive symptoms who do not respond well to antipsychotics, and are at higher risk of developing MTF. Our findings suggest NS in adolescents, alongside other factors including ethnicity, family history and neurodevelopmental comorbidity may delineate “hard to treat” subgroups. These groups may benefit from more careful monitoring and quicker access to additional interventions beyond antipsychotic medication.^53^ Follow-up was conducted for the sample for up to 5 years, so it is important to understand that antipsychotic medication may still successfully reduce positive psychotic symptoms in these groups, but NS and other MTF risk factors may moderate the association between positive symptom reduction and the protective factors required for a sustained remission. Our findings also highlight the need for research involving agents that work on alternative pathophysiological pathways (eg, the glutamate system) which may be of greater relevance to these subgroups, given their potential effectiveness at treating both the NS and the positive symptoms of those with psychosis.

Our findings support the notion that NS are intrinsic to EOP (across different psychosis diagnostic categories) and are already present during the first psychotic break. In regard to the prevalence across the different psychosis disorder classifications, in our sample NS were present in about one-third of all EOP diagnostic subgroups, with slightly higher rates in those with non-affective psychosis. This suggests that in EOP, differences between psychosis diagnostic categories (especially between schizophrenia and affective psychoses) are quantitative rather than qualitative in nature, and all diagnoses are associated with presence of impairing symptoms (as reflected by similar rates of NS). Further research using transdiagnostic approaches, as illustrated in this study, are needed to advance in the understanding of the physiopathology and predictive value of NS across disorders.

The main strengths of this study include the use of a large historical cohort of first-episode EOP, which provides a “real world” sample of young people accessing inpatient and outpatient first episode psychosis CAMH services. Selecting an early-onset sample at first episode, reduces the potential bias incurred through unknown treatment exposures. The large sample size, and relative long duration of assessment provides sufficient power to estimate the association between NS and MTF even after adjustment for a number of potential clinical confounders, including psychotic disorder classification, family history, positive symptoms, substance misuse, neurodevelopmental and depressive disorder comorbidity. Using a clinical rater review of the whole EHR for sub-sets of patients allowed us to compute performance estimates of the different text extraction tools used in the study and select the most accurate ones, and mitigation of misclassification errors. This work using text mining approaches for NS identification in large scale naturalistic samples of EOP using EHRs serves to complement the more traditional approaches using selective cohorts and intensive structured assessments, to inform prognostic indicators in clinical practice. It is important to recognize that even the most accurate NLP applications will be limited by the text held within clinical records, and unlikely to identify NS as accurately as specialized rating scales. However, as with most structured psychiatric assessments, clinicians tend to shun structured templates or drop-down options when keeping a record of their daily practice,^54,55^ so the free-text note persists as the predominant method of recording clinical information.^56^ This was certainly reflected in our EOP samples, as we were unable to detect any young people who had undergone a comprehensive assessment for NS using a standardized instrument at first presentation.

Results derived from the EOP sample should be interpreted in the context of several limitations, some of which have been covered in previous work.^29^ In relation to the findings specific to this study, it was difficult to ascertain whether extracted NS were primary or secondary in nature, we assume that as NS were rated early (ie, within 60 d of presentation to services and potentially prior or at the point of starting initial antipsychotic treatment), and excluding the presence of psychomotor retardation from the total NS counting, the NS we detect are mainly (but not only) primary in character. In regard to the MTF definition, we were unable to obtain relevant antipsychotic data such as maximum daily antipsychotic dose, antipsychotic serum levels, or structured assessments of tolerability, which may have provided more objective assessments of treatment failure. Besides, by rating treatment failure to 1 of 4 potential categories at each point of discontinuation/treatment failure, we may have underestimated the contribution of other underlying reasons to treatment failure. As with all observational studies, our findings may be limited by residual confounding, eg, we were unable to adjust for the duration of untreated psychosis—which could be explanatory factors for older age being associated with MTF. Another related limitation includes the restriction of age to the clinical samples, so that all clinical outcomes occurred prior to age 18. One of the reasons we imposed this was to reduce the impact of clinician heterogeneity as a residual confounder. Children with long-term conditions, such as psychosis, experience very different treatment environments when they move from CAMHS to adult psychiatric services,^57^ and this heterogeneity may have considerable influence on the way clinical data is recorded, as well as the mental health treatments offered and outcomes obtained.^58^ Finally, there is a chance that not all children and adolescents experiencing a first-episode psychosis within the catchment area (who access clinical services) would have presented to SLaM CAMHs, nor given potential changes in residence away from SLaM services, were all young peoples’ psychiatric care captured by the health record system over the course of follow-up. Given the mean duration of follow-up was lower in the NS group, we suspect that this may have led to an underestimation of the NS-MTF effect we report. Furthermore, the impact of potential loss to follow-up or of non-actual first presentation to services is likely to be limited, as we conducted a sensitivity analyses which took account of residence within the local catchment which showed little difference from whole sample findings.

In summary, our study demonstrated that there is a high prevalence of NS in EOP around patients’ first presentation to services and across psychosis diagnosis classifications. The finding supports the hypothesis that presence of these symptoms around the first stages of the illness identify a subset of children and adolescents who may be at higher risk of responding poorly to antipsychotics, both through refractory symptoms and high sensitivity to side-effects. Optimization of current pharmacological and non-pharmacological strategies for these patients, and further research involving agents that better target NS are warranted.

## Funding

J.D. received supported by a Medical Research Council (MRC) Clinical Research Training Fellowship (MR/L017105/1) and Psychiatry Research Trust Peggy Pollak Research Fellowship in Developmental Psychiatry. H.D. and S.L. have received salary support from the Foundation of Professional Services to Adolescents, UK. R.D.H. was funded by an MRC Fellowship (MR/J01219X/1). R.P. was funded by an MRC CRTF (MR/K002813/1). C.A., L.P-C., and C.M.D-C. have held grants from the Spanish Ministry of Economy, Industry and Competitiveness. Instituto de Salud Carlos III, co-financed by ERDF Funds from the European Commission, “A way of making Europe,” CIBERSAM, Madrid Regional Government (S2010/BMD-2422 AGES), European Union Structural Funds and European Union Seventh Framework Program under grant agreements FP7-HEALTH-2009-2.2.1-2-241909 (EU-GEI), FP7-HEALTH-2009-2.2.1-3-242114 (OPTiMISE), FP7-HEALTH-2013-2.2.1-2-603196 (PSYSCAN)and FP7- HEALTH-2013-2.2.1-2-602478 (METSY); European Union H2020 Program under the Innovative Medicines Initiative 2 Joint Undertaking (grant agreement No-115916; PRISM); Fundación Alicia Koplowitz and Fundación Mutua Madrileña. M.H., J.H.M. and H.S. receive salary support from the National Institute for Health Research (NIHR) Biomedical Research Centre at South London and Maudsley NHS Foundation Trust and King’s College London. The views expressed are those of the authors and not necessarily those of the NHS, the NIHR or the Department of Health.

## Supplementary Material

Supplementary Material

## References

[1] Díaz-Caneja CM, Pina-Camacho L, Rodríguez-Quiroga A, Fraguas D, Parellada M, Arango C Predictors of outcome in early-onset psychosis: a systematic review. NPJ Schizophr. 2015;1:14005. 27336027 10.1038/npjschz.2014.5 PMC4849440

[2] World Health Organisation. The ICD-10 Classification of Mental and Behavioural Disorders: Clinical Descriptions and Diagnostic Guidelines. Geneva, Switzerland: World Health Organisation; 1992.

[3] Schimmelmann BG, Conus P, Cotton S, McGorry PD, Lambert M Pre-treatment, baseline, and outcome differences between early-onset and adult-onset psychosis in an epidemiological cohort of 636 first-episode patients. Schizophr Res. 2007;95:1–8. 17628441 10.1016/j.schres.2007.06.004

[4] Stentebjerg-Olesen M, Pagsberg AK, Fink-Jensen A, Correll CU, Jeppesen P Clinical characteristics and predictors of outcome of schizophrenia-spectrum psychosis in children and adolescents: a systematic review. J Child Adolesc Psychopharmacol. 2016;26:410–427. 27136403 10.1089/cap.2015.0097

[5] Strauss JS, Carpenter WT Jr., Bartko JJ The diagnosis and understanding of schizophrenia. Part III. speculations on the processes that underlie schizophrenic symptoms and signs. Schizophr Bull. 1974;11:61–69. 10.1093/schbul/1.11.61 4469362

[6] Tandon R, Nasrallah HA, Keshavan MS Schizophrenia, “just the facts” 4. clinical features and conceptualization. Schizophr Res. 2009;110:1–23. 19328655 10.1016/j.schres.2009.03.005

[7] Bobes J, Arango C, Garcia-Garcia M, Rejas J; CLAMORS Study Collaborative Group Prevalence of negative symptoms in outpatients with schizophrenia spectrum disorders treated with antipsychotics in routine clinical practice: findings from the CLAMORS study. J Clin Psychiatry. 2010;71:280–286. 19895779 10.4088/JCP.08m04250yel

[8] Patel R, Jayatilleke N, Broadbent M Negative symptoms in schizophrenia: a study in a large clinical sample of patients using a novel automated method. BMJ Open. 2015;5:e007619. 10.1136/bmjopen-2015-007619 PMC4577949 26346872

[9] Álvarez-Jiménez M, Gleeson JF, Henry LP Road to full recovery: longitudinal relationship between symptomatic remission and psychosocial recovery in first-episode psychosis over 7.5 years. Psychol Med. 2012;42:595–606. 21854682 10.1017/S0033291711001504

[10] Austin SF, Mors O, Secher RG Predictors of recovery in first episode psychosis: the OPUS cohort at 10 year follow-up. Schizophr Res. 2013;150:163–168. 23932664 10.1016/j.schres.2013.07.031

[11] Milev P, Ho BC, Arndt S, Andreasen NC Predictive values of neurocognition and negative symptoms on functional outcome in schizophrenia: a longitudinal first-episode study with 7-year follow-up. Am J Psychiatry. 2005;162:495–506. 15741466 10.1176/appi.ajp.162.3.495

[12] White C, Stirling J, Hopkins R Predictors of 10-year outcome of first-episode psychosis. Psychol Med. 2009;39:1447–1456. 19187566 10.1017/S003329170800514X

[13] Kirkpatrick B, Buchanan RW, Ross DE, Carpenter WT Jr A separate disease within the syndrome of schizophrenia. Arch Gen Psychiatry. 2001;58:165–171. 11177118 10.1001/archpsyc.58.2.165

[14] Parellada M, Castro-Fornieles J, Gonzalez-Pinto A Predictors of functional and clinical outcome in early-onset first-episode psychosis: the child and adolescent first episode of psychosis (CAFEPS) study. J Clin Psychiatry. 2015;76:e1441–e1448. 26580481 10.4088/JCP.13m08863

[15] Strauss GP, Allen DN, Miski P, Buchanan RW, Kirkpatrick B, Carpenter WT Jr Differential patterns of premorbid social and academic deterioration in deficit and nondeficit schizophrenia. Schizophr Res. 2012;135:134–138. 22130110 10.1016/j.schres.2011.11.007 PMC3288761

[16] Rapado-Castro M, Soutullo C, Fraguas D Predominance of symptoms over time in early-onset psychosis: a principal component factor analysis of the positive and negative syndrome scale. J Clin Psychiatry. 2010;71:327–337. 20331934 10.4088/JCP.08m04845yel

[17] Chang WC, Tang JY, Hui CL The relationship of early premorbid adjustment with negative symptoms and cognitive functions in first-episode schizophrenia: a prospective three-year follow-up study. Psychiatry Res. 2013;209:353–360. 23473654 10.1016/j.psychres.2013.02.014

[18] Hassan GA, Taha GR Long term functioning in early onset psychosis: two years prospective follow-up study. Behav Brain Funct. 2011;7:28. 21801438 10.1186/1744-9081-7-28 PMC3162891

[19] Vyas NS, Hadjulis M, Vourdas A, Byrne P, Frangou S The Maudsley early onset schizophrenia study. Predictors of psychosocial outcome at 4-year follow-up. Eur Child Adolesc Psychiatry. 2007;16:465–470. 17896122 10.1007/s00787-007-0621-4

[20] Stewart R, Davis K ‘Big data’ in mental health research: current status and emerging possibilities. Soc Psychiatry Psychiatr Epidemiol. 2016;51:1055–1072. 27465245 10.1007/s00127-016-1266-8 PMC4977335

[21] Daniel DG Issues in selection of instruments to measure negative symptoms. Schizophr Res. 2013;150:343–345. 23899996 10.1016/j.schres.2013.07.005

[22] Fields JH, Grochowski S, Lindenmayer JP Assessing positive and negative symptoms in children and adolescents. Am J Psychiatry. 1994;151:249–253. 8296898 10.1176/ajp.151.2.249

[23] Kay SR, Fiszbein A, Opler LA The positive and negative syndrome scale (PANSS) for schizophrenia. Schizophr Bull. 1987;13:261–276. 3616518 10.1093/schbul/13.2.261

[24] Kapila A, Fisher HL, Johnson S Clinical and demographic differences between patients with manic, depressive and schizophrenia-spectrum psychoses presenting to early intervention services in London [published online ahead of print October 15, 2017]. Early Interv Psychiatry. doi:10.1111/eip.12511. 10.1111/eip.12511 29034588

[25] Tasma M, Swart M, Wolters G Do routine outcome monitoring results translate to clinical practice? A cross-sectional study in patients with a psychotic disorder. BMC Psychiatry. 2016;16:107. 27091333 10.1186/s12888-016-0817-6 PMC4836179

[26] Chowdhury GG Natural language processing. Annu Rev Inf Sci Technol. 2003;37:51–89.

[27] Liao KP, Cai T, Savova GK Development of phenotype algorithms using electronic medical records and incorporating natural language processing. BMJ. 2015;350:h1885. 25911572 10.1136/bmj.h1885 PMC4707569

[28] Murphy SN, Weber G, Mendis M Serving the enterprise and beyond with informatics for integrating biology and the bedside (i2b2). J Am Med Inform Assoc. 2010;17:124–130. 20190053 10.1136/jamia.2009.000893 PMC3000779

[29] Downs JM, Lechler S, Dean H The association between comorbid autism spectrum disorders and antipsychotic treatment failure in early-onset psychosis: a historical cohort study using electronic health records [published online ahead of print November 7, 2017]. J Clin Psychiatry. doi:10.4088/JCP.16m11422 10.4088/JCP.16m11422 PMC6037287 29125721

[30] Demjaha A, Egerton A, Murray RM Antipsychotic treatment resistance in schizophrenia associated with elevated glutamate levels but normal dopamine function. Biol Psychiatry. 2014;75:e11–e13. 23890739 10.1016/j.biopsych.2013.06.011

[31] Laruelle M Schizophrenia: from dopaminergic to glutamatergic interventions. Curr Opin Pharmacol. 2014;14:97–102. 24524997 10.1016/j.coph.2014.01.001

[32] Robinson DG, Woerner MG, Alvir JM Predictors of treatment response from a first episode of schizophrenia or schizoaffective disorder. Am J Psychiatry. 1999;156:544–549. 10200732 10.1176/ajp.156.4.544

[33] Boeing L, Murray V, Pelosi A, McCabe R, Blackwood D, Wrate R Adolescent-onset psychosis: prevalence, needs and service provision. Br J Psychiatry. 2007;190:18–26. 17197652 10.1192/bjp.190.1.18

[34] Downs J, Gilbert R, Hayes RD, Hotopf M, Ford T Linking health and education data to plan and evaluate services for children. Arch Dis Child. 2017;102:599–602. 28130218 10.1136/archdischild-2016-311656 PMC5519948

[35] Perera G, Broadbent M, Callard F Cohort profile of the South London and maudsley NHS foundation trust biomedical research centre (SLaM BRC) case register: current status and recent enhancement of an electronic mental health record-derived data resource. BMJ Open. 2016;6:e008721. 10.1136/bmjopen-2015-008721 PMC4785292 26932138

[36] Cunningham H, Tablan V, Roberts A, Bontcheva K Getting more out of biomedical documents with GATE’s full lifecycle open source text analytics. PLoS Comput Biol. 2013;9:e1002854. 23408875 10.1371/journal.pcbi.1002854 PMC3567135

[37] Jackson MSc RG, Ball M, Patel R, Hayes RD, Dobson RJ, Stewart R TextHunter–A user friendly tool for extracting generic concepts from free text in clinical research. AMIA Annu Symp Proc. 2014;2014:729–738. 25954379 PMC4420012

[38] Downs J, Hotopf M, Ford T Clinical predictors of antipsychotic use in children and adolescents with autism spectrum disorders: a historical open cohort study using electronic health records. Eur Child Adolesc Psychiatry. 2016;25:649–658. 26472118 10.1007/s00787-015-0780-7 PMC4889626

[39] Hayes RD, Downs J, Chang CK The effect of clozapine on premature mortality: an assessment of clinical monitoring and other potential confounders. Schizophr Bull. 2015;41:644–655. 25154620 10.1093/schbul/sbu120 PMC4393681

[40] Schneider C, Papachristou E, Wimberley T Clozapine use in childhood and adolescent schizophrenia: a nationwide population-based study. Eur Neuropsychopharmacol. 2015;25:857–863. 25769917 10.1016/j.euroneuro.2015.02.003

[41] Suzuki T, Remington G, Mulsant BH Defining treatment-resistant schizophrenia and response to antipsychotics: a review and recommendation. Psychiatry Res. 2012;197:1–6. 22429484 10.1016/j.psychres.2012.02.013

[42] Kahn RS, Fleischhacker WW, Boter H; EUFEST study group Effectiveness of antipsychotic drugs in first-episode schizophrenia and schizophreniform disorder: an open randomised clinical trial. Lancet. 2008;371:1085–1097. 18374841 10.1016/S0140-6736(08)60486-9

[43] Lieberman JA, Stroup TS, McEvoy JP; Clinical Antipsychotic Trials of Intervention Effectiveness (CATIE) Investigators Effectiveness of antipsychotic drugs in patients with chronic schizophrenia. N Engl J Med. 2005;353:1209–1223. 16172203 10.1056/NEJMoa051688

[44] Patel R, Wilson R, Jackson R Association of cannabis use with hospital admission and antipsychotic treatment failure in first episode psychosis: an observational study. BMJ Open. 2016;6:e009888. 10.1136/bmjopen-2015-009888 PMC4785290 26940105

[45] Marder SR, Davis JM, Chouinard G The effects of risperidone on the five dimensions of schizophrenia derived by factor analysis: combined results of the North American trials. J Clin Psychiatry. 1997;58:538–546. 9448657 10.4088/jcp.v58n1205

[46] McLennan D, Barnes H, Noble M, Davies J, Garratt E, Dibben C. The English Indices of Deprivation 2010. London, UK: Department of Communities and Local Government 2011.

[47] Jackson RG, Patel R, Jayatilleke N, Natural language processing to extract symptoms of severe mental illness from clinical text: the Clinical Record Interactive Search Comprehensive Data Extraction (CRIS-CODE) project BMJ Open 2017;7:e012012. doi: 10.1136/bmjopen-2016-012012 10.1136/bmjopen-2016-012012 PMC5253558 28096249

[48] Shaffer D, Gould MS, Brasic J A children’s global assessment scale (CGAS). Arch Gen Psychiatry. 1983;40:1228–1231. 6639293 10.1001/archpsyc.1983.01790100074010

[49] Sarkar S, Hillner K, Velligan DI Conceptualization and treatment of negative symptoms in schizophrenia. World J Psychiatry. 2015;5:352–361. 26740926 10.5498/wjp.v5.i4.352 PMC4694548

[50] Perivoliotis D, Morrison AP, Grant PM, French P, Beck AT Negative performance beliefs and negative symptoms in individuals at ultra-high risk of psychosis: a preliminary study. Psychopathology. 2009;42:375–379. 19752591 10.1159/000236909

[51] Millan MJ, Fone K, Steckler T, Horan WP Negative symptoms of schizophrenia: clinical characteristics, pathophysiological substrates, experimental models and prospects for improved treatment. Eur Neuropsychopharmacol. 2014;24:645–692. 24820238 10.1016/j.euroneuro.2014.03.008

[52] Howes OD, Kapur S A neurobiological hypothesis for the classification of schizophrenia: type A (hyperdopaminergic) and type B (normodopaminergic). Br J Psychiatry. 2014;205:1–3. 24986384 10.1192/bjp.bp.113.138578

[53] Hunter R, Barry S Negative symptoms and psychosocial functioning in schizophrenia: neglected but important targets for treatment. Eur Psychiatry. 2012;27:432–436. 21602034 10.1016/j.eurpsy.2011.02.015

[54] Gilbody SM, House AO, Sheldon TA Psychiatrists in the UK do not use outcomes measures. National survey. Br J Psychiatry. 2002;180:101–103. 11823316 10.1192/bjp.180.2.101

[55] Zimmerman M, McGlinchey JB Why don’t psychiatrists use scales to measure outcome when treating depressed patients?J Clin Psychiatry. 2008;69:1916–1919. 19192467 10.4088/jcp.v69n1209

[56] Roberts A Language, structure, and reuse in the electronic health record. AMA J Ethics. 2017;19:281–288. 28323609 10.1001/journalofethics.2017.19.3.stas1-1703

[57] Campbell F, Biggs K, Aldiss SK Transition of care for adolescents from paediatric services to adult health services. Cochrane Database Syst Rev. 2016;4:CD009794 27128768 10.1002/14651858.CD009794.pub2 PMC10461324

[58] Green J Annotation: the therapeutic alliance–a significant but neglected variable in child mental health treatment studies. J Child Psychol Psychiatry. 2006;47:425–435. 16671926 10.1111/j.1469-7610.2005.01516.x

