# Supplementary material for: Negative Symptoms in Early-Onset Psychosis and Their Association With Antipsychotic Treatment Failure
Source: Schizophr Bull. 2018 Jan 24;45(1):69–79. doi: 10.1093/schbul/sbx197 (PMC6293208; doi:10.1093/schbul/sbx197)
Supplement: Supplementary Material [file sbx197_suppl_supplementary_material.doc]

**Supplementary Material 1: Summary of negative and positive symptom items extracted from the EHR** at first presentation to services in early-onset psychosis subjects

|  |  | **Total sample (N= 638)** | | | | **MTF (N=124)** | | **Example text for NLP extraction** | |
| --- | --- | --- | --- | --- | --- | --- | --- | --- | --- |
| **Symptom categories** | **N(%)** | | **Total items detected** | **Items detected**  **mean (S.D, IQR)** | **N(%)** | **Total items detected** | **Items detected**  **mean (S.D, IQR)** | **Positive annotation** | **Negative annotation** |
| **Negative items** |  | |  |  |  |  |  |  |  |
| Blunted affect | 130 (20.3) | | 457 | 0.72 (1.6, 9) | 29 (23.4) | 147 | 1.18 (2.3, 10) | His affect remains very blunted | incongruent affect, Stable affect |
| Emotional  withdrawal | 214 (33.5) | | 848 | 1.33 (2.3, 12) | 54 (43.6) | 258 | 2.10 (2.9, 11) | mother described her as becoming withdrawn, not communicating; | not sad or withdrawn during the assessment |
| Poor rapport | 62 (9.7) | | 176 | 0.28 (0.9, 6) | 18 (14.5) | 61 | 0.49 (1.2, 5) | He had poor rapport, very limited rapport | We established a good rapport |
| Social isolation | 51 (8.0) | | 226 | 0.35 (1.3, 8) | 14 (11.3) | 88 | 0.71(2.3, 6) | He withdrew socially from friends and family, | no evidence of being socially withdrawn |
| Poverty of speech | 32 (5.0) | | 84 | 0.13 (0.6, 4) | 8 (6.5) | 17 | 0.13 (0.6, 4) | He has poverty of speech | Speech normal, no pressure or poverty of speech apparent |
| Mutism | 66 (10.3) | | 462 | 0.72 (3.6, 28) | 25 (20.2) | 203 | 1.67(5.6, 28) | She has periods of 'mutism' | Mother denied he had any periods of being mute |
| ≥ 2 NS | 239 (37.5) | | - | - | 65 (52.4) | - | - |  |  |
| **Positive items** |  | |  |  |  |  |  |  |  |
| Delusions | 277 (43.4) | | 1145 | 1.8 (4.6, 2) | 74 (60) | 449 | 3.62 (8.6, 3) | continued to express delusional ideas | No evidence of delusions, |
| Hallucinations | 483 (76.4) | | 5226 | 8.2 (12.5, 18) | 103 (83.0) | 1761 | 14.2 (18.4, 17 ) | visual distortions and auditory hallucinations | No evidence of responding to any hallucinations |
| Paranoid ideation | 427 (66.9) | | 3579 | 5.61 (11.9, 6) | 94 (75.8) | 1237 | 10.0 (21.2, 10) | He said the neigbours cause him to feel paranoid | No appearance of paranoia, denied paranoid feelings |
| Persecutory ideation | 180 (28.4) | | 540 | 0.84 (2.3, 1) | 45 ( 36.3) | 152 | 1.22 (3.3, 1) | She suffered persecutory delusion | He denied any worries of persecution |

Note: EHR: Electronic Healthcare Record; IQR: interquartile range; MTF: multiple treatment failure; NLP: natural language processing; NS: negative symptoms; S.D: Standard Deviation

**Supplementary Material 2: Reasons for multiple treatment failure in young people with early-onset psychosis, with and without negative symptoms at first presentation**

|  | N (%) of individuals^b^ | |
| --- | --- | --- |
| Reasons for MTF^a^ | Non - NS  (*n* = 41) | NS  (*n* = 50) |
| Persistent insufficient response | 6 (14.6) | 7 (14.0) |
| Persistent adverse effects | 9 (21.9) | 10 (20.0) |
| Persistent non-adherence | 2 (4.9) | 3 (6.0) |
| Variability in reasons |  |  |
| - Insufficient response and adverse effects | 11 (26.9) | 21 (42.0) |
| - Insufficient response and non-adherence | 3 (7.3) | 4 (8.0) |
| - Adverse effects and non-adherence | 10 (24.4) | 5 (10.0) |
| ^a^ Comparison in reasons for MTF between Non-NS and NS groups; fisher exact test, p=0.49  ^b^ In all cells, % refers to percentages (within columns) of individuals for whom information on main reason of discontinuation was available (n=91). Excluded due to no reason ' or 'other reason' ascertained were: Non-NS n= 18 (31%); NS group n=15 (23%)  Note: MTF: multiple treatment failure; NS: negative symptoms | | |

| **Supplementary Material 3: Baseline characteristics profile at first presentation and association with multiple treatment failure over time in early-onset psychosis (n=618)** | | | |
| --- | --- | --- | --- |
| **Baseline characteristics** | Non MTF Sample | MTF sample | Crude H.R. (95% CI) |
| **≥2 baseline Marder NS** | 167 (33.7) | 49 (46.2) | **1.98 (1.35 – 2.91)**** |
| **Female gender** | 238 (48.0) | 57 (53.8) | 1.12 (0.76-1.65) |
| **Mean age at referral (s.d)** | 15.6 (1.9) | 15.3 (1.7) | **1.30 (1.13-1.50) ***** |
| **Ethnicity, N(%)** |  |  |  |
| White | 243 (48.9) | 43 (40.6) | Reference |
| Black | 150 (30.2) | 44 (41.5) | 1.64 (1.08-2.49)* |
| Asian | 30 (6.1) | 6 (5.7) | 1.31 (1.30-3.03) |
| Mixed | 56 (11.3) | 13 (12.3) | 1.45 (0.77-2.69) |
| Not Stated | 17 (3.4) | 0 (0) | - |
| **Neighbourhood Characteristics, N(%)** ^a^ |  |  |  |
| 1^st^ (Least Deprived) | 121 (25.1) | 34 (33.0) | Reference |
| 2^nd^ | 118 (24.4) | 23 (22.1) | 0.69 (0.41-1.18) |
| 3^rd^ | 124 (24.7) | 21 (20.6) | 0.64 (0.37-1.11) |
| 4^th^ (Most Deprived) | 120 (24.8) | 24 (23.5) | 0.66 (0.39-1.12) |
| **First ICD-10 psychosis diagnosis,** **N(%)** |  |  |  |
| Other Psychoses^b^ | 81 (16.3) | 21 (19.8) | Reference |
| Bipolar Disorder / F30, F31 | 32 (6.5) | 9 (8.5) | 1.26 (0.57-2.76) |
| Drug-induced psychosis / F1x.x5 | 29 (5.9) | 4 (3.8) | 0.80 (0.27-2.34) |
| Schizophrenia / F20 | 293 (59.1) | 52 (49.1) | 0.79 (0.47-1.31) |
| Schizoaffective / F25 | 7 (1.4) | 7 (6.6) | **3.10 (1.31-7.33)**** |
| Psychotic Depression / F32.3, F33.3 | 54 (10.9) | 13 (12.3) | 1.05 (0.52-2.10) |
| **Co-morbid neuropsychiatric disorders, N(%)** |  |  |  |
| Autism Spectrum Disorder | 81 (16.3) | 29 (27.4) | 1.24 (0.80-1.91) |
| Other neurodevelopmental disorder | 80 (16.1) | 16 (15.1) | **0.72 (0.43-1.23)** |
| Major Depressive Disorder | 142 (28.6) | 26 (26.4) | 0.82 (0.53-1.27) |
| **First degree relative with psychotic disorder** | 97 (19.6) | 33 (31.3) | **1.84 (1.22-2.77)**** |
| **Illness severity/ Functioning** |  |  |  |
| Admission at presentation, N(%) | 176 (35.5) | 54 (50.9) | **2.33 (1.59-3.43)***** |
| CGAS score (mean, SD)^b^ | 39.3 (15.8) | 36.2 (15.8) | **0.985 (0.971-0.999)*** |
| **Positive symptom** |  |  |  |
| 1^st^ (lowest quartile) | 57 (11.5) | 9 (8.5) | Reference |
| 2^nd^ | 92 (18.5) | 11 (10.4) | 0.89 (0.36 – 2.15) |
| 3^rd^ | 183 (36.9) | 31 (29.3) | **1.35 (0.64-2.84)** |
| 4^th^ (highest quartile) | 164 (30.1) | 55 (51.9) | **2.42 (1.19-4.89)*** |
| **Substance misuse** |  |  |  |
| Cannabis | 219 (44.2) | 44 (41.5) | 1.22 (0.83-1.80) |
| Cocaine or crack | 84 (16.4) | 14 (13.2) | 0.94 (0.53-1.64) |
| Amphetamines | 16 (3.3) | <5 (<5) | 1.17 (0.32-3.71) |
| MDMA | 14 (2.8) | <5 (<5) | 0.51 (0.07-3.69) |

**p* < .05; ***p* < .01; ****p* < .001; % Refers to percentages within columns, for whom information was available

^a^ Missing cases =19; ^b^ Data available in a subsample of 384, ^b^ Other Psychoses: an ICD-10 diagnosis of ‘brief psychotic disorder (F23)’, ‘delusional disorder (F22), ‘shared psychotic disorder’, or ‘psychosis not otherwise specified (NOS)’.Note: CGAS: Children’s Global Assessment Scale; MTF: multiple treatment failure; NS: negative symptoms;
